# Determining the Latent Tuberculosis Infection by IFN - γ Elispot Assay in Healthcare Workers From University Hospitals of Shiraz, South West of Iran

**DOI:** 10.5812/ircmj.3635

**Published:** 2013-06-05

**Authors:** Mohammad Taheri, Hamid Bazrafkan, Mojtaba Habibagahi

**Affiliations:** 1 Department of Microbiology, School of Paramedical Sciences, Shiraz University of Medical Sciences, Shiraz, IR Iran; 2 Immunotherapy laboratory, Department of Immunology, School of Medicine, Shiraz University of Medical Sciences, Shiraz, IR Iran

**Keywords:** Latent tuberculosis, Tuberculin test, Interferon - gamma, Enzyme-Linked Immunospot Assay, Iran

## Abstract

**Background:**

Classical screening methods are incapable to properly detect LTBI (Latent TB Infection) and HCWs (Healthcare Workers) are at the high risk of exposure. Only few reports estimated the prevalence of LTBI among Iranian HCWs and they mostly used the TST (Tuberculin Skin Test), rather than assessing the response against TB-specific antigens.

**Objectives:**

The current study aimed to determine the frequency of IFN - γ producing blood cells of microbiology and radiology ward technicians by an in-house IFN - γ ELISPOT assay in the University hospitals of Shiraz University of Medical Sciences (SUMS) against recombinant ESAT - 6 and PPD antigens.

**Materials and Methods:**

89 HCWs from medical laboratory and radiology departments of Shiraz University of Medical Sciences’ hospitals, South of Iran, were screened for LTBI. To achieve the goal, an in-house IFN - γ (Interferon - gamma) ELISPOT (Enzyme Linked ImmunoSpot) assay was used to detect the reactivity against ESAT - 6 (Early Secreted Antigen Target protein - 6) and the PPD (Purified Protein Derivate).

**Results:**

Almost 8% of the personnel showed positive TST (over 10 mm) reaction while 29% of them had considerable T - cell reactivity against PPD in ELISPOT assays. However, the ESAT - 6 reactivity was found only in one case of HCWs. No correlation was found between the patterns of the reactions and the age or the duration of the employment or previous vaccination history of the participants. The ELISPOT results were not correlated with the TST results.

**Conclusions:**

Considering the hindrance of TST, the IFN - γ ESAT - 6 ELISPOT assay, even in forms of in-house tests, could replace traditional methods to properly spot the LTBI among the high risk groups from Iran’s health system.

## 1. Background

Tuberculosis (TB) is a health problem which globally causes two million deaths every year. Disease is the result of the infection with bacteria of Tuberculosis complex comprising *M. tuberculosis*, *M. bovis* and *M. africanum*. Other mycobacteria, known as atypical forms, such as *M. avium* species can also cause disease or diagnostic problems (1, 2), Due to the nature of the tuberculosis infection, fast and accurate diagnosis is an advantage and very important element of community’s health to control the disease. After so many years culture is still the most sensitive available diagnostic test as its sensitivity rates up to 98%. However, it takes up to 6 - 8 weeks to identify a *M. tuberculosis* isolate from a clinical specimen and in many occasions the bacillus could not be successfully cultured. Only a minority of cases may develop active disease after TB infection while the majority of cases stay unidentified in a state of LTB (Latent Tuberculosis) Infection and spread the disease. Therefore, spotting these individuals not only provides the chance of treatment, but also halts the dissemination of the bacteria to new hosts. TST (Tuberculin skin test), based on the detection of delayed type hypersensitivity reaction to the antigenic mixture of PPD, is currently the most popular test to detect latent TB infection. In addition to patients’ compliance problem, presence of shared antigens in PPD and possible boosting the immune response by repeated tests, there have been several occasions of false positive or uncertain results in TST (3, 4). However, exploitation of newer methods, known as IFN - γ release assays (IGRAs), is less affected by external factors and could identify active and/or latent TB patients according to IFN - γ production against mycobacterial antigens(5). Of so many mycobacterial antigens, the ELISPOT, (ESAT-6) and the culture filtrate protein 10, (CFP10) of RD1 region of bacterial genome are identified as two major antigens which are absent in BCG and most environmental mycobacteria (6-8). These antigens are able to stimulate T cells for IFN - γ production (9, 10) and could form the basis of blood tests that could overcome most disadvantages of TST (11), to distinguish the LTBI (Latent Tuberculosis Infection ) even in endemic developing countries (12, 13). IGRA based commercial tests (QuantiFeron-TB Gold; Cellestis Ltd., St. Kilda, Australia and T SPOT-TB; Oxford Immunotec, Oxford, UK) have shown a positive result in most cases with a high likelihood of latent TB infection and were negative in healthy BCG vaccinated individuals (14-20). Most data regarding LTBI in Iran are based on TST. According to the World Health Organization, the estimated incidence of TB infection in Iran is 28 cases per 100,000 individuals (21). Amongst population, healthcare workers and laboratory technicians are more exposed to infectious agents which urges health care systems to adapt new available strategies for rapid diagnosis of TB infection.

## 2. Objectives

The current study prepared an in-house IFN - γ ELISPOT assay and determined the frequency of IFN - γ producing blood cells of microbiology and radiology ward technicians in the University hospitals of Shiraz University of Medical Sciences (SUMS) against recombinant ESAT - 6 and PPD antigens.

## 3. Materials and Methods

### 3.1. Participants

Employees from clinical microbiology laboratories and radiology departments at five hospitals in Shiraz-Iran were prospectively recruited during November 2009 to February 2010. Eighty nine eligible employees agreed to participate in the study and answered questionnaires concerning TB and their job history. All the participants had at least one year history of full time work in the medical laboratories or radiology departments. Neither of the selected participants had the experience of hospital admission for TB or other severe pulmonary diseases. According to the periodical medical records of the personnel, no participant had evidence of HIV infection and no one took immunosuppressive therapies at the time of the study. The study was approved by the institutional review board of the University Ethics Committee and all the participants gave their written informed consent accordingly.

### 3.2. Tuberculin Skin Test

TST was administered by the Mantoux method at the time of interview, after blood sampling. A volume of 100 µl of PPD antigen mixture (equal to 10 tuberculin units) (Razi vaccine and serum production Institute, Iran) was inoculated by intra-dermal injection into the forearm. The skin reaction of induration was measured after 48 and then 72 hours with a ruler and recorded in millimeters. The results were read independently and blinded by two experienced staffs. For interpretation of the TST induration, a 10 mm cutoff was considered to define positive results.

### 3.3. Peptide Antigens

Recombinant *M. tuberculosis* antigen ESAT - 6 (Statens Serum Institute, Denmark) and PPD antigen mixture (Razi Vaccine and serum institute, Iran) were used in IFN - γ ELISPOT assays. The homodimer ESAT - 6 antigen of 196 amino acid residues and 20.5 Dalton molecular weight was the product of *Lactococcus lactis*. The PPD antigen mixture was concentrated by vacuum concentrator (Heto Drywinner, Denmark), dialyzed overnight against PBS (pH7.2) with multiple changes and its total protein content was adjusted for use in T cell activation assays.

### 3.4. ELISPOT Assay

For IFN - γ ELISPOT assays, the PVDF membrane of the culture plates (Millipore, UK) was treated with 70% ethanol and rinsed with PBS. Diluted anti-IFN - γ antibody (U - Cytech, Netherlands) was loaded into the wells and incubated overnight at 4 ºC. Peripheral blood mononuclear cells (PBMCs) from the volunteers were prepared by blood centrifugation over Ficoll Hypaque density gradient (Biosera, UK). The purified cells were re-suspended in RPMI culture media supplemented with 10% FCS and antibiotics and cell counts were adjusted. A total of 2X10^5^ PBMCs in 100 µl were seeded into Ab-coated wells and stimulated in duplicates with ESAT - 6 or PPD antigens at 5 µg/ml and 15 µg/ml, respectively. PBMCs in the culture medium alone or stimulated with phytohemagglutinin (PHA) (Gibco, UK) at 2.5 µg/ml were used as negative and positive controls, respectively. The plates were kept still for 20 hours at 37°C with 5% CO2 and 96% humidity. After the incubation time, the cells were washed off from the wells and the presence of IFN - γ producing cells was revealed by reagents from U-Cytech (Netherlands), according to the manufacturer’s instruction. Positive reactions as black spots were analyzed after drying the wells. The number of spot-forming units (SFU) in each well was counted manually with dissection microscope (SZH10 Olympus, Japan) and digitally photographed. Counting was performed by two independent observers and recorded individually. The responses were scored as positive when the test well contained ≥ 2 SFU than the negative control wells or had at least twice as many SFU as the negative control wells. Responses were classified as independent and re-tested again if the number of spots in the positive control wells was less than 20 or the number of spots in the negative controls was more than five.

### 3.5. IFN - γ ELISA Assay

The replicate cultures of PBMCs, as described before, were stimulated with ESAT - 6 and incubated for forth days. The culture supernatants in duplicates were harvested and the aliquots were kept frozen at -80 ºC. IFN - γ contents in the supernatants were measured by high sensitivity (0.06 pg/ml) commercial ELISA (Bendermed Systems, Austria).

### 3.6. Statistical Analysis

Mann-Whitney test was used to show the differences between data from different groups. Spearman’s test was used to evaluate the correlation between the findings. Agreement between the results of the tuberculin skin test and ELISPOT assays was assessed by ƙ coefficients. In those analyses, kappa values less than 0.4 indicated a weak agreement; values of 0.41 - 0.60 indicated good agreement and values above 0.6 showed strong agreement. P value less than 0.05 (proportionally divided to the number of comparing groups) was considered as significantly different in all analyses. SPSS version 11.5 (SPSS, Chicago, IL, USA) and Graphpad prism version 4 (Graphpad Inc; San Diego CA, USA) were used for statistical analyses and data presentations.

## 4. Results

### 4.1. Demographic Results

To detect latent TB infection among 89 healthcare workers from five hospitals of SUMS, we screened their T cells’ reactivity against ESAT - 6 and compared that with PPD reaction. Table 1 summarizes demographic information of the participants. We recruited 26 male and 24 female medical laboratory technicians plus 22 male and 17 female personnel of radiology departments. The mean age of the participants was 30.7 years. The duration of employment as healthcare worker of the recruited technicians was between 1 - 33 years (Mean ± SD: 7.47±7.96), each technician works at least for 40 hours per week. Neither of the selected staff showed chronic cough or other suspicious symptoms of tuberculosis. All the participants were immunized with BCG vaccination in the past and three times acid fast staining of the sputum was reported negative for all the samples. The results of the in vivo and in vitro tests were not significantly different among both sexes of the participants. 

**Table 1.** Characteristics of 89 Healthcare Workers Studied for LTBI

	No	Frequency
**Gender**		
Women	41	46
Men	48	54
**Age**		
23 - 29	57	64
30 - 39	18	20.2
40 - 49	10	11.2
50 - 59	2	2.3
> 60	2	2.3
**Years of Work as Healthcare worker**		
1 - 4	53	59.5
5 - 9	14	15.7
10 - 14	8	9
15 - 24	9	10.1
> 25	5	5.6
**BCG Vaccination**		
Yes	89	100
No	0	0
**TST**		
Positive	7	7.9
Negative	82	92.1
**History of active TB**		
Yes	0	0
No	89	100

### 4.2. TST Results

The tuberculin skin test was performed by Mantoux method and analyzed after 48 and then 72 hours. The diameter of the indurations ranged between 3 - 30 mm in the tested participants; however, only seven volunteers (7.86%) had skin reactions greater than or equal to 10 mm. There was no significant correlation between the skin test results and the duration of employment (r= -0.16, p = 0.137). 

### 4.3. IFN - γ ELISPOT Results

**Figure 1.**
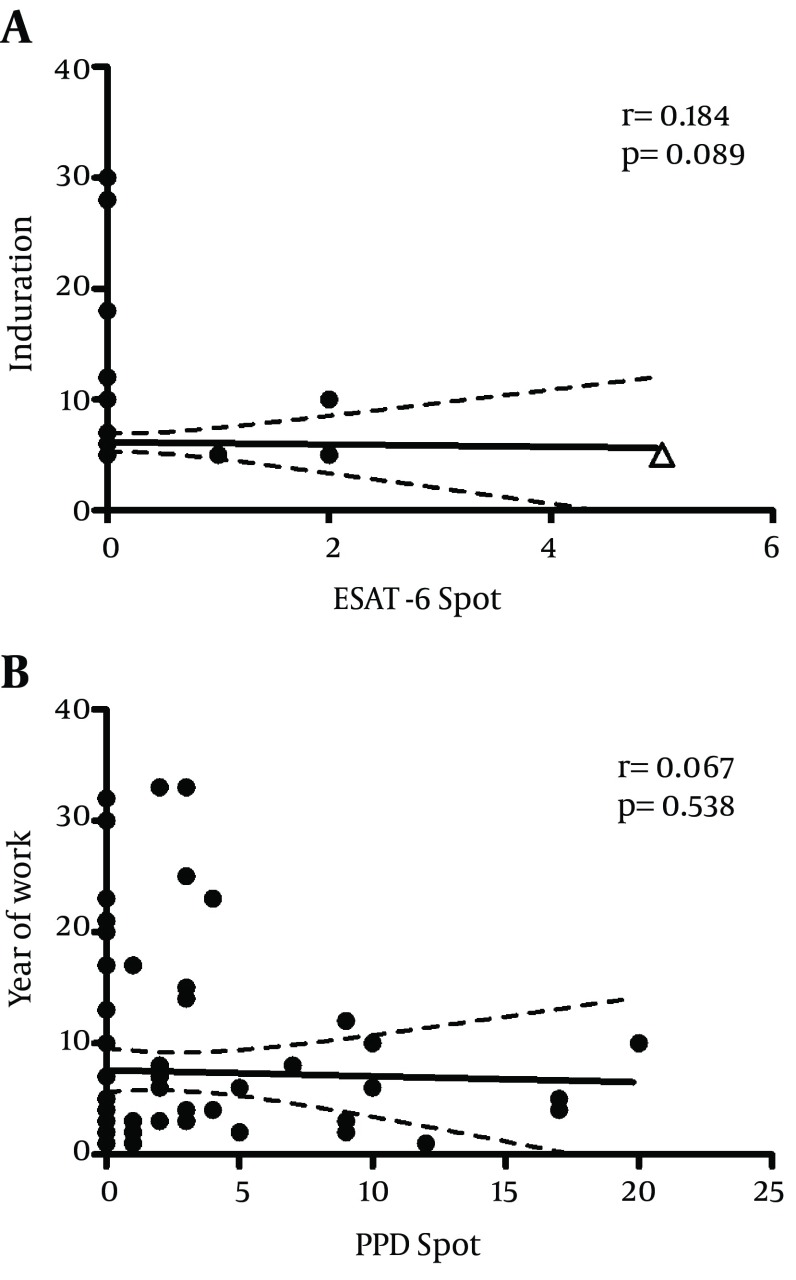
Correlation of in vitro and in vivo Responses of 89 SUMS Health Care Workers Against PPD

**Figure 2.**
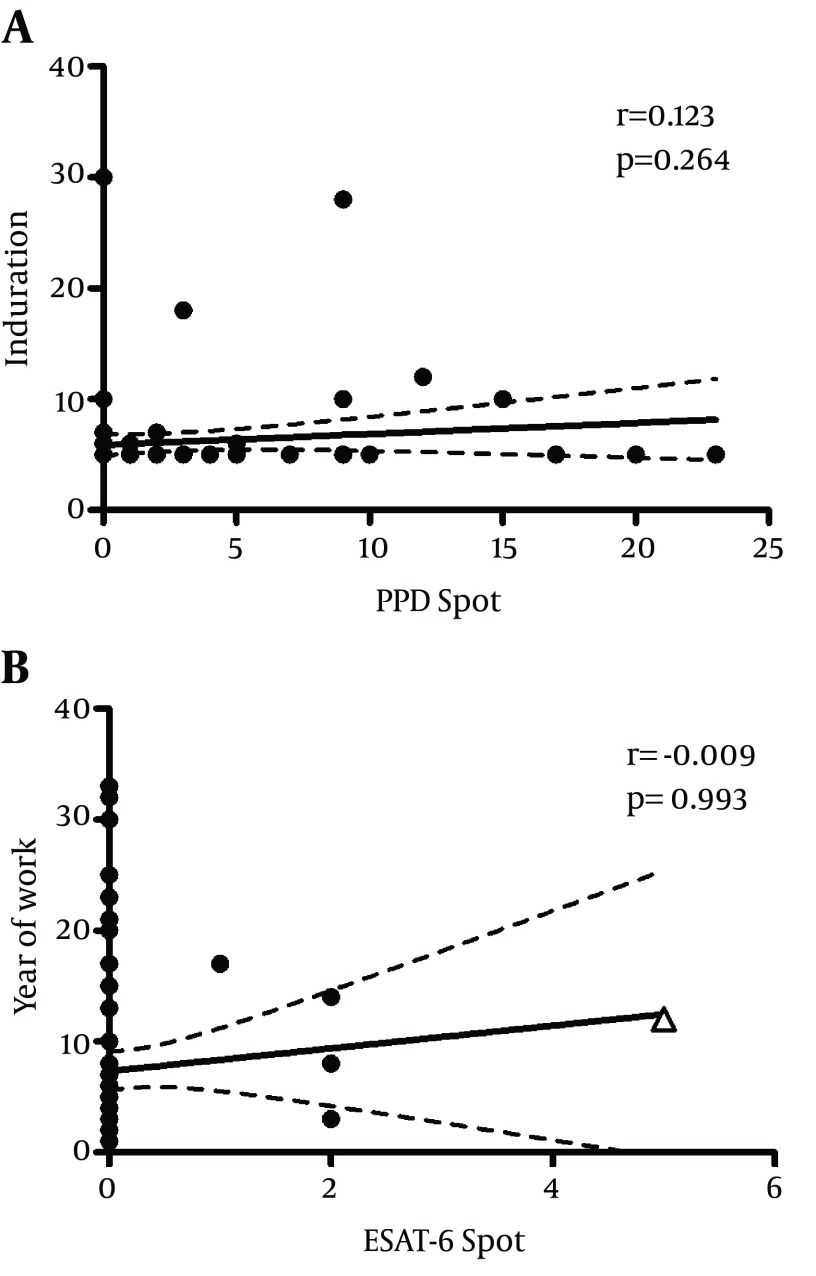
Correlation of in vitro and in vivo Responses of 89 SUMS Health Care Workers Against ESAT-6 Antigen.

Recombinant ESAT - 6 peptide and purified protein derivative (PPD) antigen mixture were used to stimulate mycobacteria specific memory T cells from healthcare personnel during an overnight culture in an in-house IFN - γ ELISPOT assay. The spots in duplicate reactions showing the presence of Ag-specific IFN - γ producing cells were screened, photographed and recorded. All negative control wells contained no Ag stimulation had ≤ 2 spots. Of 89 healthcare workers screened in this study, only one laboratory technician (1.1%) was defined as positive by IFN - γ ESAT - 6 assay showed 5 SFU (more than negative control) along 2X10 ^5 ^cultured PBMCs. Meaningful positive anti-PPD reactivity was seen in at least 26 cases (29.2%). The maximum frequency of PPD-specific IFN - γ secreting cells in the tested individuals was up to 23 SFU in 2X10 ^5 ^PBMCs. The results showed a significant difference between the pattern of reactivity against PPD and ESAT - 6 in the tested individuals (p < 0.0001). Statistical examination of data, using Spearman analysis, did not demonstrate significant correlation of PPD IFN-γ ELISPOT reactivity with TST results (r = 0.123, p = 0.264) (Figure 1 A) or the duration of employment as healthcare worker (r = 0.067, p = 0.538) (Figure 1 B). Similarly, ESAT - 6 IFN - γ ELISPOT reactivity correlated with neither of those two parameters (r = 0.184, p = 0.089 and r = -0.009, p = 0.993, respectively), (Figure 2 A and Figure 2 B). Analyzing the whole data showed lack of statistical agreement between the ESAT - 6 and PPD ELISPOT tests with TST results (ƙ-values of 0.194 and 0.262, respectively). 

The single case with positive ESAT - 6 IFN - γ ELISPOT test was a 36 year old laboratory technician with a history of 12 years of work. He also showed positive PPD ELISPOT reactivity while his TST induration measurement was negative. He was referred to the center for infectious disease control for further investigations.

## 

## 5. Discussion

Due to job responsibilities, healthcare workers are always at the high risk of exposure to different infectious sources or can act as a potential source of infection and spread the disease (22), In this context, healthcare workers represent an important target group for latent tuberculosis infection screening programs. Therefore, in the current study, as the first report, the extent of the latent *M. tuberculosis* infection was estimated in a selected group of the healthcare workers of the major SUMS hospitals by a sensitive in-house IFN - γ ELISPOT assay using ESAT-6 as the specific antigen stimulant and it was compared to anti-PPD reactivity and TST. Analyzing the results from 89 eligible volunteers showed the presence of only one person (1.1%) with positive IFN - γ ESAT - 6 reactivity whereas higher numbers of the tested personnel showed reactions against PPD re-stimulation in vitro and in vivo. Such new techniques, exploiting TB-specific antigens, might better differentiate immune responses to mycobacteria, distinguishes true infected people and demonstrates the width of the infection among the population and people in higher risks. Utilizing new techniques are simple, fast and feasible to do. The performance and the validation of the in-house made IFN - γ ELISPOT assay was checked in a series of different studies where the authors blindly screened several groups of individuals (TB patients of different stages and healthy volunteers) which showed acceptable differentiation efficiency (manuscript in preparation). ESAT – 6 was used rather than its combination with CFP10 which is mostly used in the commercial tests. Using 10 mm cut off point, the TST revealed positive in up to 8% of participants. Surprisingly, 8% positive TST reactivity is far less than the reports from healthcare workers in some other countries such as India, Germany or Switzerland, where the BCG vaccination is also common (23-25) . The 8% positive rate is in accordance with other reports from Iran, showing that the TST positive rate in nursing is between 2% - 14% (26). The IFN - γ ELISPOT PPD reactivity (positive in 29.2% of participants) did not correlate with the tuberculin skin test results which use the same antigen. However, neither of the tested participants with positive TST reaction or PPD reactivity in this study showed ESAT - 6 responsive cells in ELISPOT assays. This would suggest that the majority of positive TST reactivity could be false positive reaction against antigens rather than *M. tuberculosis*. Therefore, such persons can be saved from unnecessary tuberculosis treatments by testing against more TB specific antigens. Interestingly, the tuberculin skin test result of the person with positive ESAT - 6 reactivity, and the history of BCG vaccination in the past, was negative. Again it means that the TST could not spot the right person with possible LTBI and might mislead to mark healthy personnel for TB infection. The purified PBMCs was also re-stimulated with ESAT - 6 antigen for four days in duplicated cultures. The quantity of IFN - γ secretion in neither of the cultures from healthcare workers was enough to be measured in a sensitive ELISA test and all the samples turned negative, even the person with positive ESAT - 6 IFN - γ ELISPOT reactivity. Using the same technique, the authors were able to show the production of various amounts of IFN - γ by PBMCs from a series of patients with tuberculosis and differentiate them from healthy individuals (data not shown). It may show the necessity of higher amounts of cytokine production to be detected in ELISA systems while ESAT-6 IFN - γ ELISPOT reactivity could detect and enumerate the small numbers of *m. tuberculosis* reactive cells and show higher sensitivity. Only five SFU over the background, were detected for the positive HCW. In spite of this, literature shows that the whole blood ELISA for TB patients could show a similar sensitivity as of ELISPOT (27). Considering the new trend for assuming ESAT - 6 IFN - γ ELISPOT results as indication of latent tuberculosis infection, one can observe much lower incidence of infected personnel among healthcare workers in the SUMS hospitals than it is estimated in the general population by TST results. It should be remembered that molecular analysis of tuberculosis infection shows that newly infected cases in Iran have a minor role in the disease dissemination and the majority of the detected active cases of the tuberculosis are resulted from reactivation of old infections.(28) Therefore, replacement of tuberculin skin test with IFN - γ ELISPOT assay using *M. tuberculosis* specific antigens and re-evaluation of LTBI in general population and high risk groups should eliminate a large proportion of those false positive reactions, increase the accuracy of patient screening and save a significant amounts of funds with better results. 
